# Slow science: the value of long ocean biogeochemistry records

**DOI:** 10.1098/rsta.2013.0334

**Published:** 2014-09-28

**Authors:** Stephanie A. Henson

**Affiliations:** National Oceanography Centre, European Way, Southampton SO14 3ZH, UK

**Keywords:** marine science, ocean biogeochemistry, ocean biology, sustained observations, climate change

## Abstract

Sustained observations (SOs) have provided invaluable information on the ocean's biology and biogeochemistry for over 50 years. They continue to play a vital role in elucidating the functioning of the marine ecosystem, particularly in the light of ongoing climate change. Repeated, consistent observations have provided the opportunity to resolve temporal and/or spatial variability in ocean biogeochemistry, which has driven exploration of the factors controlling biological parameters and processes. Here, I highlight some of the key breakthroughs in biological oceanography that have been enabled by SOs, which include areas such as trophic dynamics, understanding variability, improved biogeochemical models and the role of ocean biology in the global carbon cycle. In the near future, SOs are poised to make progress on several fronts, including detecting climate change effects on ocean biogeochemistry, high-resolution observations of physical–biological interactions and greater observational capability in both the mesopelagic zone and harsh environments, such as the Arctic. We are now entering a new era for biological SOs, one in which our motivations have evolved from the need to acquire basic understanding of the ocean's state and variability, to a need to understand ocean biogeochemistry in the context of increasing pressure in the form of climate change, overfishing and eutrophication.

## 1. Introduction

Sustained observations (SOs) of ocean biogeochemistry have a rich history extending back over 50 years [1,2]. Increased pressure on funding agencies in recent times has led to enhanced scrutiny of the role for SOs within the panoply of ocean biogeochemical research. At the same time, an increasing recognition of the need to understand the impacts on ocean biogeochemistry of current and future climate change brings with it the obligation to adequately monitor the Earth system. Thus, this Theme Issue on a ‘Prospectus for UK Marine Sustained Observations’ is timely. This article was solicited by the Challenger Society for Marine Science and the UK Scientific Committee on Oceanic Research and represents my personal perspective on the value and future development of SOs for biogeochemical research.

Biological SOs date back to the early days of routine monitoring, when the original platforms were weather ships, typically deployed in far-flung parts of the world ocean to gather data designed to improve weather forecasts. For example, in 1949 Harald Sverdrup collected biological and physical data over the course of a year from Weather Ship M in the Norwegian Sea, which led to his seminal work on the conditions necessary to initiate a spring bloom [3]. The establishment in the 1950s of time-series stations dedicated specifically to the study of oceanography, e.g. Hydrostation S in the Sargasso Sea and Ocean Station PAPA in the Northeast Pacific, heralded the next stage in the history of open-ocean SOs. Although these time-series stations, regularly sampled by research vessels, were originally established to understand how meteorological forcing affects seasonal and interannual variability in hydrography, the value of regular, repeated sampling soon became evident to a broader range of scientists. This encompassed an emerging interest in fisheries management and the recognition that regular monitoring could assist in improved management of stocks. Complementary programmes were soon added to investigate, for example, the flux of sinking material, nutrient cycling and the interactions between trophic levels. At about the same time, an early pioneer in the field of biological SOs, the Continuous Plankton Recorder (CPR) survey, was established, providing spatially and temporally resolved information on plankton abundance.

Numerous additional SOs have been established in the intervening 50 years, many of which have been able to maintain near-continuous observations. The advent of new technologies for autonomous and remote ocean sampling have always driven SOs forward, so that time-series stations are now joined by a veritable arsenal of observing systems: satellite ocean colour, moorings, automated Ferrybox and Ship of Opportunity systems, gliders and profiling floats. The motivation for SOs has also changed since their inception, although more subtly than the technology has, from a desire to acquire basic understanding of the ocean's state and variability, to a more nuanced need to understand this variability in the context of increasing pressure on our oceans in the form of climate change, overfishing and eutrophication.

Carbon dioxide (CO_2_) emissions continue to rise at a rate of approximately 2.5–3% year on year [4], which is at the upper end of projected emissions in Intergovernmental Panel on Climate Change (IPCC) scenarios [5]. Details of the ocean's role in modulating the atmospheric inventory of CO_2_ and feedback effects on marine biota remain uncertain. The predicted effects of climate change include an increase in stratification, which would further limit the vertical supply of nutrients, which in subtropical regions could result in decreased primary production [6]. The oligotrophic gyres are thus expected to expand, further reducing global productivity. In subpolar regions, increased stratification may result in an earlier alleviation of light limitation, extending the phytoplankton growth season to earlier in the year [6]. Changing temperatures also directly affect many physiological processes, and metabolic rates generally increase with increasing temperature [7]. Phytoplankton community structure is expected to shift towards dominance by smaller functional types that thrive in warm, relatively nutrient-poor waters [8]. This could have knock-on effects for nutrition and food availability for higher trophic levels, including fish stocks. Additionally, smaller phytoplankton are less efficient at exporting carbon from the surface to the deep ocean [9], thus altering food supply to the mesopelagic and benthic fauna. However, there is some evidence that the range of N_2_-fixing organisms may increase with enhanced stratification [10] and can contribute significantly to sinking organic carbon flux [11]. Rising temperatures would also increase remineralization in the mesopelagic zone, further reducing the amount of carbon transferred to the deep ocean and resulting in a positive feedback loop with atmospheric CO_2_ concentration [12].

The increase in atmospheric CO_2_ also leads directly to ocean acidification, by increasing the concentration of dissolved CO_2_ (a weak acid) in seawater. Ocean pH is currently approximately 0.1 units lower than in the pre-industrial era and is forecast to decrease by a further 0.2–0.3 units by 2100 [13]. Many organisms with a carbonate substrate, e.g. coccolithophores, foraminifera and corals, have shown a reduction in calcification rates under experimental high-CO_2_ conditions, although there have also been several laboratory studies that showed no response or a positive response to artificial ocean acidification [14]. Laboratory and field experiments also demonstrate that the photosynthetic rates of non-calcifying organisms do not respond to increased CO_2_ conditions and N_2_-fixing organisms may exhibit increased rates of carbon and nitrogen fixation under elevated CO_2_ [14].

An additional effect of climate change on ocean biology is deoxygenation, which is driven by the decreased solubility of gas in warmer water and a reduction in ventilation due to increased stratification. Model predictions indicate a reduction in the ocean's dissolved oxygen content of approximately 1–7% by 2100 [7], and, although the global mean oxygen concentration remains above anoxic levels, some regions become hypoxic or suboxic. For some periods and locations, predicted oxygen concentration drops below 20 μmol kg^−1^, which is lethal for most higher marine organisms [15].

These predictions for the response of ocean biology to climate change are based on predictive models, themselves developed from our conceptual understanding of the interactions between atmospheric and oceanic forcing and the marine ecosystem. This understanding owes its existence, in part, to SOs, which provided the multi-year measurements of seasonal and interannual variability necessary to pick apart the roles of forcing and response. Many of our predictions about the response of ocean biogeochemistry to climate change are based on using the observations of contemporary variability as an analogue for future responses. For example, we may observe that, over several years, warmer-than-average summers lead to lower-than-average phytoplankton abundance, and we may extrapolate from that observation to suggest that climate change will result in reduced phytoplankton populations. SOs have provided the means by which we can first develop such hypotheses and then test them (provided of course that time series of observations are maintained). These observations of forcing and response, and repeat measurements of biological processes and parameters, have enabled the development and validation of biogeochemical models, including global coupled models run under climate change scenarios, which are included in the IPCC assessment for the first time in 2014 (to be published on www.ipcc.ch). Maintaining SOs into the future is critical for monitoring ocean biology and biogeochemistry, and detecting future changes in response to climate warming, in addition to continuing to provide fundamental insights into the functioning of ocean biogeochemistry.

In this paper, I focus on open-ocean SOs with a substantial biogeochemistry component. Companion papers in this Theme Issue cover the UK and Ireland's coastal SOs [16] and open-ocean physical oceanography-centred SOs [17].

## 2. The unique perspective offered by sustained observations

The persistence of many SOs over multiple decades, despite constant threats from funding squeezes, pays testament to the value of the data obtained from them. The unique perspective that SOs offer centre principally around one theme: variability. SOs provide invaluable information on the temporal (and, in some cases, spatial) variability in ocean biogeochemistry on a wide range of time scales, from the sub-seasonal to the decadal. Quantifying the characteristics of this variability is crucial for several reasons: firstly, to elucidate the controls on a particular parameter or process (i.e. if we do not know how it changes, we cannot figure out what is affecting it, or what effect it has); secondly, to understand the typical range of variability (i.e. a handful of observations cannot tell us what is ‘normal’); and finally, to identify more readily an anomalous event or long-term trend (i.e. without a baseline, we have no way of identifying what is ‘unusual’). A final benefit of many SOs is that they provide a focal point for concerted community effort, often on multi-disciplinary topics. The ‘build it and they will come’ philosophy [1,18] has borne fruit, owing to the clear benefits of exploiting pre-existing infrastructure where core measurements are reliably sampled. The resulting reduced costs, combined with the accumulation of data and knowledge of the oceanography and biogeochemical functioning of a region, have facilitated many studies (specific examples of ancillary studies that used SO as a platform are detailed in later sections).

Variability is ubiquitous in the biological properties of the ocean, which makes the interpretation of data from a single cruise or small set of observations extremely tricky. Have we captured a true representation of the process of interest? Or were the measurements affected by some unusual event? And how would we know anyway? Variability is sometimes perceived as a problem in interpreting oceanographic data, and indeed it can be if only a handful of observations are available. However, thanks to the time series supplied by SOs, variability becomes a ‘window’ into understanding what controls a particular parameter or process. By making repeat observations over the course of a year, or over several years, the relationships between forcing and response may be revealed.

Because ocean biogeochemistry is highly variable on a multitude of time scales, establishing what is ‘normal’ for a particular location would be nigh on impossible without repeated, sustained observations. Multiple years of data are required to characterize the typical range in seasonal amplitude and timing of events. It seems obvious to modern oceanographers with the benefit of hindsight that just 1 or 2 years of data are insufficient to determine a typical seasonal cycle or characterize interannual variability. However, it is worth reminding ourselves here of how this understanding arose: through SOs, which, by providing long time series, permitted us initially to comprehend, and finally to quantify, the large variability in ocean biogeochemistry.

The ability to establish a baseline of what is ‘normal’ is also critical to our ability to recognize an unusual event or long-term trend, e.g. to separate natural variability from anthropogenic forcing. This requires long time series—many years to decades, depending on the process of interest. For example, to identify a particular year as ‘normal’ or ‘anomalous’, a decade or more of observations is needed, covering a large range of possible forcing conditions (e.g. warm years and cool years). If the response to a decadal climate oscillation, such as the North Atlantic Oscillation (NAO) or Pacific Decadal Oscillation (PDO), is of interest, then 20–40 years of data may be necessary to characterize the conditions of a positive and a negative phase [19,20]. For detecting climate change-driven trends in primary production, 30–40 years of data (or more) are needed to distinguish the natural variability on interannual to decadal time scales from the long-term trend [21]. Although the length of time series needed to define the baseline, and thus identify anomalies, depends strongly on location (because some regions have strong natural variability, others weak), it is clear that SOs maintained over multiple decades are absolutely key to understanding the operation of the ocean's biogeochemistry and its susceptibility to change.

## 3. Key breakthroughs from sustained observations

**Figure 1.**
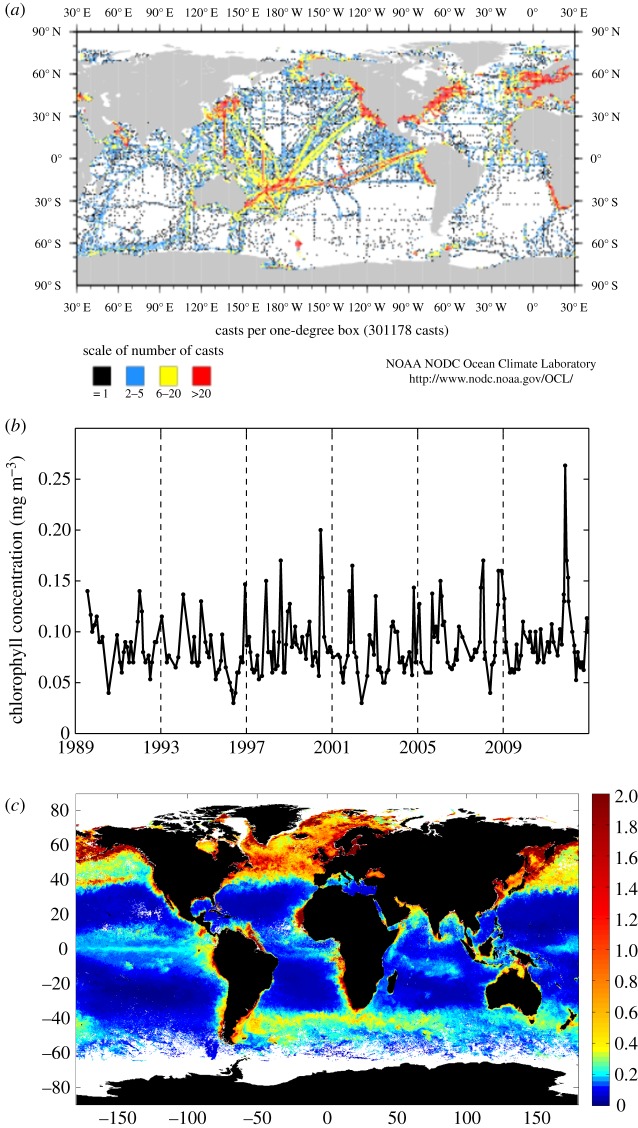
(*a*) Density of *in situ* surface chlorophyll concentration measurements collected between 1773 and 1997, extracted from the World Ocean Database at http://www.nodc.noaa.gov/OC5/SELECT/dbsearch/dbsearch.html; data collected from time-series stations, shipboard profiles and under-way systems, and gliders. (*b*) *In situ* chlorophyll concentration from the top 10 m collected at the Hawaii Ocean Time-series site every two to four weeks between 1989 and 2012; acquired from http://hahana.soest.hawaii.edu/hot/hot-dogs/. (*c*) Global chlorophyll concentration (mg m^−3^) at 9 km spatial resolution averaged between April and June 1998 acquired by the SeaWiFS satellite; data downloaded from http://oceancolor.gsfc.nasa.gov/. (Online version in colour.)

In the past 30 years or so, SOs have wrought a revolution in biological oceanography. To put this into perspective, I present an example based on chlorophyll concentration. Between 1773 and 1997, chlorophyll was measured *in situ* at a total of approximately 300 000 stations (as reported in the World Ocean Database). Although this is a huge number of data points, inspection of the data distribution in figure 1*a* shows that large parts of the ocean have less than five measurements and some have none at all. Vast tracts of the Southern Ocean, for example, have zero or only one data point in the database. It would be difficult to ascertain even the broad-scale distribution of chlorophyll concentration from this dataset, and one would struggle to deduce any information on temporal variability on the large scale. The advent of regular sampling at SO locations began to reveal detailed information about the seasonal (and eventually interannual) variability in chlorophyll concentration, as shown in the example for the Hawaii Ocean Time-series site (HOT; figure 1*b*). Information on the temporal variability of chlorophyll allowed significant advances in our understanding of the range of variability and the physical and biological factors that control chlorophyll concentrations. However, time-series station data cannot provide any information on the spatial variability in chlorophyll, except at the largest scales (e.g. the differences between ocean basins through a comparison of HOT and the Bermuda Atlantic Time-series Study site; BATS). The arrival of satellite-derived chlorophyll data in the late 1970s prompted another revolution in biological oceanography as the dramatic spatial variability in chlorophyll on mesoscales was revealed (figure 1*c*) and the provision of high-resolution, global images of chlorophyll on a daily basis led to step changes in our understanding of, for example, physical–biological interactions, mesoscale processes and the effect of climate oscillations, such as El Niño–La Niña (see [22] for a review). Each of these daily satellite chlorophyll images contains approximately 3 million non-cloudy pixels, i.e. we are now acquiring 10 times more chlorophyll measurements every day than were obtained in the entire 200 years prior to the advent of ocean colour satellites.

Although satellite-derived chlorophyll data occasioned an order-of-magnitude increase in the quantity of data, they have not negated the need for *in situ* SOs, firstly to provide the calibration and validation data to ensure the quality of satellite algorithms, and secondly because there are many properties that cannot be derived from satellite or other remotely sensed observations. Indeed, there is a growing appreciation of how the synthesis of remotely sensed data, *in situ* observations and model studies can elucidate key processes in biological oceanography.

This rapid increase in the amount of data available from SOs has enabled some significant breakthroughs in biological oceanography. Here, I highlight four areas where I believe that SOs have made the key contributions to our current understanding. For each highlighted area, space constraints allow only a handful of results from many possible examples to be described, but hopefully these serve to illustrate the diversity of science being generated by SOs, both from UK programmes and worldwide.

### (a) Variability

The widespread introduction of SOs allowed the first glimpses into the extraordinarily large seasonal and interannual variability in ocean biogeochemistry. The issue of variability in SO datasets has in turn vexed and benefited users, who on the one hand face issues such as separating temporal from spatial variability, and on the other can take the opportunity to investigate interactions between ocean physics and biology.

The fundamental role of SOs in driving our understanding of variability is illustrated by the example of the El Niño phenomenon. El Niño events in 1972 and 1976 prompted the establishment in 1982 of an SO programme of tri-weekly sampling at shore stations off Peru and the Galapagos Islands and quarterly shipboard transects. This proved to be prescient, as a large El Niño developed in 1982/1983. The repeated physical, chemical and biological measurements allowed aspects of the earlier conceptual models of El Niño effects to be tested with observations for the first time. In the case of ocean biogeochemistry, increased sea surface temperature resulted in nitrate concentrations and primary production that were lower than ‘presumably normal’ [23]. Note the need to preface this observation with ‘presumably’; a lack of quantitative time-series data made it difficult to define ‘normal’, emphasizing again one of the key benefits of SOs. The anomalous nature of the biological situation and link to decreased populations of seabirds, mammals and commercial fish species was difficult to establish owing to the lack of phytoplankton time-series data prior to 1982. The Tropical Atmosphere Ocean array was then established in 1994, although biological sensors were not added until 1997, at the same time that the ocean colour instrument, SeaWiFS, was launched. This again proved to be fortuitous, as a very strong El Niño followed by a La Niña occurred almost immediately. The basin-scale satellite data provided for the first time an insight into the Pacific-wide biological response to El Niño events. It was immediately recognized that ‘the description and explanation of these dynamic changes would not have been possible without an observing system that combines biological, chemical and physical sensors … with remote sensing of chlorophyll’ [24], p. 2126.

The same sentiment holds true for our understanding of biology's response on sub-seasonal to decadal time scales to many other forcings—whether a local-scale response to mesoscale features [25] or changes in wind mixing or heating [26], or a basin-scale response to a shift in the phase of a major climate oscillation [27,28]. In UK waters and across the North Atlantic, the CPR dataset has led the way in defining the basin-scale response of lower trophic levels to interannual and decadal variability [29]. The NAO and Atlantic Multidecadal Oscillation have been linked to multi-decadal variability in phytoplankton and zooplankton abundance through changes in ocean physics [29,30,31], with corresponding knock-on effects up the food chain to fish larvae [32]. At BATS, the relationship between NAO phase and zooplankton biomass, via the intermediary steps of changing temperature, stratification and primary production, has been clarified thanks to decades of repeat observations [33]. In the Pacific, SOs have established that the phase of the PDO is linked to the timing of the phytoplankton bloom [34] and changes in both the phytoplankton and zooplankton community structure via changes in ocean circulation [35]. The links between changes in stratification, nutrient fluxes, primary production and plankton assemblages driven by climatic variability, including El Niño events, have been elucidated by studies at HOT [36,37]. In short, SOs have enabled tremendous progress in elucidating physical–biological interactions [38], basin-wide differences in response to similar basin-wide forcing [39] and long-term variability in ecosystem structure [40].

A multitude of additional examples from throughout the world's oceans could be included here, but hopefully this small subset of results illustrates the revolution in our knowledge of the biological response to natural variability that SOs have enabled. Without long time series of repeated measurements, we simply would not know what is normal and what is not.

### (b) Trophic dynamics

One of the most influential studies in biological oceanography stems from the earliest days of SOs based at weather ships. Harald Sverdrup's observations during the spring of 1949 of water column properties and the phytoplankton population led to his seminal ‘critical depth hypothesis’ to explain the conditions leading to the onset of the spring bloom [3]. Refinement of Sverdrup's theory has continued since, including the use of years of data from a coastal station (L4 in coastal waters close to Plymouth Sound, UK) to suggest that some phytoplankton species can bloom by escaping control by microzooplankton through predator avoidance strategies [41]. Argo floats equipped with bio-optical sensors have also been used to address the concurrent changes in physics and chlorophyll concentrations in the transition to bloom conditions [42]. The ability to make repeated observations through an entire seasonal cycle is one of the primary advantages of an SO and which continues to be exploited to provide insights into the interactions within and between different trophic levels of the marine ecosystem.

Another classic conceptual model that SOs were central in developing and testing is the ‘match–mismatch’ hypothesis [43], which states that prey availability during critical periods of predator development controls their survival, so that mismatches in the timing of the phytoplankton bloom and larval spawning can later affect adult populations. Studies combining SOs of satellite chlorophyll concentration and fishery surveys have found that delayed phytoplankton blooms can affect the survival of haddock juveniles [44] and the timing of shrimp egg hatch [45]. In the North Sea, decades of data from the CPR survey revealed that the timing of the peak abundance of different functional groups of phyto- and zooplankton changed at different rates so that a mismatch in trophic linkages occurred [46].

Multiple years of SO data enable the full range of trophic links to be investigated; for example, at BATS the relationships spanning the size range from bacterioplankton to zooplankton have been established [47]. Similarly, at HOT, the repeated sampling has allowed the interactions between nano- and picoplankton populations, and the coupling to mesozooplankton, to be investigated [48]. SOs at Long Term Ecological Research stations in the West Antarctic Peninsula found that the dominant grazer in the food web switched between salps and krill on interannual time scales [49]. This kind of detailed information on trophic interactions and food web structure would be difficult to capture without repeated observations over many seasons and years, only possible from SO platforms.

SO programmes are often a focal point for the oceanographic community, and so process studies are frequently based around them that do not necessarily require the repeated nature of SOs, but nevertheless lead to key discoveries. For example, the Atlantic Meridional Transect (AMT) programme has played host to many ‘visiting scientists’ on its cruises, resulting in improved understanding of the role of the ocean's smallest denizens. Small phytoplankton had been thought to provide a constant background level of primary production, but data collected on AMT cruises demonstrated that much of the variability in low-productivity regions is driven by nanoplankton [50], and indeed nanoplankton can contribute 30–50% of carbon fixation [51]. Additionally, the depth-integrated biomass of bacteria is now understood to be equivalent to the picoplankton [52], and control of the bacterial population is by grazing by nanoplankton, themselves less abundant than picoplankton although their productivity is comparable [53]. The discovery that unicellular cyanobacteria are able to fix nitrogen and are abundant enough to play a significant role in the global nitrogen cycle also came from a project ‘piggy-backing’ on an SO's core programme, this time at HOT [54]. This handful of examples hopefully serves to illustrate the enormous breadth of understanding gained of various aspects of the marine food web through SOs, such as the importance of unicellular nitrogen fixers to nitrogen biogeochemistry, the heterotrophic nature of nanoplankton and insights into the mechanisms underlying phytoplankton bloom phenology.

### (c) Ocean biology's role in the global carbon cycle

SOs have played a central part in elucidating the role of ocean biology in the carbon cycle, from air–sea CO_2_ fluxes to deep-ocean carbon sequestration. Even small changes in the remineralization of organic carbon in the ocean can have large consequences for atmospheric CO_2_ concentrations [12], so understanding the magnitude and variability of the biological contribution to the oceanic carbon sink, and the processes that control it, is critical to assessing the current and future fate of atmospheric CO_2_.

Ships of opportunity that regularly cross the Atlantic equipped with *p*CO_2_ sensors have quantified the seasonal air–sea flux of CO_2_ and documented a reduction in the North Atlantic CO_2_ sink [55,56]. The biological drawdown of CO_2_ is driven by primary production, specifically net community production (the balance between primary production and respiration). Primary production can be estimated for the surface ocean using satellite data, although perhaps with limited reliability, at least in subtropical regions [57]. These synoptic estimates of global primary production over multiple years have revealed substantial interannual variability in global production, potentially driven by stratification changes on the large scale [58], although a recent reassessment suggests that this direct link does not hold on the local scale [59]. Variability in upper-ocean productivity has long been assumed to drive variability in carbon export into the interior; however, a paucity of time-series data of upper-ocean export makes the causal linkages hard to discern. An exception is at BATS, where repeated ship-based observations of carbon export have been used to link changes in the phytoplankton population to variability in the particulate organic carbon (POC) flux from the surface to the deep ocean [60].

The next stage of the transfer of carbon to the deep ocean, i.e. the journey of sinking particles through the mesopelagic zone, is almost entirely unsampled by SO platforms owing to technological difficulties, although promising new developments in autonomous sampling will undoubtedly eventually be able to address this data gap [61,62]. There is, however, substantial information on the quantity and quality of the organic material arriving in the deep (more than 2000 m) ocean and to the seafloor thanks to SOs. Prior to the mid-1980s, the deep ocean was considered to be a fairly unchanging environment, without significant seasonal or interannual variability. However, the discovery of bursts of sinking material arriving on the seafloor dispelled that myth [63]. Moored sediment traps also revealed a striking degree of temporal variability in deep carbon flux [64,65]. The material collected by deep traps has allowed investigation of the changing freshness, composition and organic matter content of material reaching the deep ocean, which has been linked to the efficiency of the biological carbon pump [66,67] and the biodiversity and size structure of benthic fauna [68,69]. Although some studies have made the link between variability in carbon sequestration fluxes and upper-ocean processes [11,70,71], consistent causal relationships remain elusive. Hence, our understanding of ocean biology's role in the carbon cycle, while advanced significantly by SOs, remains incomplete.

### (d) Biogeochemical modelling

Since the earliest days of biological modelling, SOs have been critical to both the development and validation of models. A series of surveys carried out at Georges Bank (off Nova Scotia) provided a handful of measurements from which in 1946 Gordon Riley was able to develop a simple model of phytoplankton biomass [72]. However, applying the same model to data from Woods Hole proved less successful [73], emphasizing the need to make repeat observations at multiple locations in order to develop robust models.

Fast forward 50 years and the data collected from SOs during the Joint Global Ocean Flux Study (JGOFS) era plus the advent of ocean colour satellites spurred on the development of sophisticated biogeochemical models that included representations of phytoplankton community structure, zooplankton grazing, carbon export and remineralization [74]. Models were able to move beyond the simple NPZD (nitrogen–phytoplankton–zooplankton–detritus) formulation, pioneered by Mike Fasham based on SO measurements [75], and include explicit representations of phytoplankton size and/or community structure [76,77], including nitrogen fixers [78] and calcifying phytoplankton [79]. As an example, data on zooplankton production and grazing rates from BATS and HOT enabled the development of more explicit phytoplankton loss terms [80]. The controls on, and fate of, organic matter exported from the upper ocean and sinking through the mesopelagic zone are still not well understood from a mechanistic standpoint, but SOs have nevertheless provided sufficient data to parametrize some of these processes [81,82].

SOs have a key role to play, not only in model development, but also in model validation. The availability of synoptic, high-resolution data from ocean colour satellites has allowed the spatial and temporal variability in global biogeochemical models to be validated [83]. *In situ* data from time-series stations are also a rich source of validation data and have led to model intercomparison studies, where the abilities and shortcomings of different parametrizations can be tested [57]. The data from the CPR survey have also been used to assess the decadal variability simulated by models and the links to climate oscillations [84].

In the past approximately 5 years, increased computing power has permitted increasingly complex models to be run at global scales. For example, a model that represents 10 specific plankton functional groups is available [85]. Trait-based models that include many tens of phytoplankton ‘ecotypes’, which are randomly assigned nutrient and light affinities from a defined range, allow studies of emergent ecosystem structure [86]. Results from these models have been validated using multiple sources of SO data, particularly from the AMT programme. Global models run in hindcast mode simulate the ocean over the past approximately 50 years and so allow exploration of observed physical–biological interactions and an extension of time-series data into the recent past. Hindcast runs have been used to investigate the response of phytoplankton productivity and bloom timing to the NAO via its influence on mixed layer depth and wind stress, thus elucidating patterns observed in the relatively short satellite ocean colour time series [87,88]. These global models have also been run under future global warming scenarios, and in 2014 biogeochemical model output will be included for the first time in the IPCC assessment (to be published at www.ipcc.ch). Multi-model ensembles of pre-industrial and future states of the ocean will permit the assessment of the response of ocean biogeochemistry to climate change.

The field of biogeochemical modelling has been immensely enriched and enabled by SOs. Without the repeated observations to define the seasonal and interannual variability in biogeochemistry, the wealth of data necessary to investigate forcing and response mechanisms, and the targeted study of specific processes at SO sites, the field of biological modelling would be very much the weaker.

## 4. What is the future of sustained observations in biogeochemical oceanography?

SOs have clearly already enabled significant advances in biological oceanography. However, there remain important future areas of investigation where SOs will be crucial. These principally centre on the response of ocean biology and biogeochemistry to ongoing climate change and its manifestations in warming, acidification and deoxygenation. In order to identify climate change-driven responses, we need to quantify the natural variability so it can be separated from any long-term trend. This necessarily requires long time series of data that adequately resolve the principal components of the natural variability (i.e. seasonal, interannual and/or decadal). For ocean primary productivity, a ‘long’ time series means of the order of 30–40 years of monthly data to distinguish a climate change trend from natural variability [21,89]. Many SO programmes are now nearing (or have exceeded) this length, implying that the time is right to begin exploiting SOs for evidence of climate change effects.

In parallel, advances in technology are enabling new processes and parameters to be measured from SO platforms. New sensors, combined with an increase in lifespan, robustness and accuracy and a decrease in size and power consumption, are permitting an ever broader range of biological variables to be used for SOs. Some existing platforms are starting to be exploited for biological SOs and need to be integrated with existing SOs, such as gliders and bio-Argo floats, and new platforms are being developed, e.g. the Carbon Flux Explorer [90] and Wave Gliders [91]. These developments go hand-in-hand with new approaches to analyse, visualize and interpret the ever-increasing quantities of data being generated by SOs.

### (a) Detecting climate change

In the Introduction, I summarized some of the predicted responses of ocean biology to climate change, including ocean warming, acidification and deoxygenation. There is now a need to determine and detect, where possible, the ongoing effects of climate warming, not only to satisfy our basic curiosity, but also to test model predictions of future change (and so refine and improve them, where necessary). The only way to address this is through analysis of the long time series of data provided by SOs. We need to assess the effect of climate change on basic biogeochemical properties (such as chlorophyll concentration, primary production, oxygen and nutrient concentrations), ecosystem indicators (phytoplankton and zooplankton community structure) and components of the global carbon cycle (air–sea CO_2_ flux, sinking flux of carbon from the surface ocean and sequestration efficiency). For some of these properties, some SOs already provide the repeated, sustained monitoring required for climate change detection, while for others the technology is still in development (see next subsection).

For all these parameters, an assessment of climate change effects requires first a quantitative understanding of the range in natural variability (and forcing factors) on time scales from seasonal to decadal (to include, for example, the response to NAO phase). Without this, it is not possible to determine whether a signal is related to natural variability or attributable to climate change. The shorter the time series available for analysis, the more acute this problem becomes. In addition, gaps in time series or changes in instrumentation, sample collection or analysis protocols can adversely affect the timely detection of climate change signals [92].

In order to advance our understanding of climate change effects on ocean biology, we need to characterize, quantify and understand contemporary seasonal, interannual and decadal variability and the biophysical interactions that drive it. This is essential to rule out misinterpreting a response to natural variability as a climate change signal. Greater understanding of forcing and response will also aid in improving model representation of ocean biology, which feeds back into helping us understand how variability arises [87,93].

The timely detection of climate change effects also requires long, consistent and gap-free time series of data, as a long time series is needed to distinguish the effects of natural variability from a long-term trend. Ideally, the time series would be sufficiently long to encompass multiple phases of any oscillatory natural variability that is experienced at a location, e.g. long enough to cover multiple El Niño–La Niña cycles or positive and negative phases of the NAO. A consistent time series is required to ensure that apparent trends or regime shifts are not due to changes in instrumentation, sampling design or analysis protocols. Either these factors must remain the same for the duration of the time series (as is the case for the CPR survey) or the effect of changing these factors on the mean and variance must be fully characterized and taken into account when investigating trends [94]. Gaps in a dataset introduce a related issue—how can one determine whether an observed change either side of a data gap is due to a long-term trend or the effect of some unobserved process? The problem is particularly acute when the gap is associated with a change in instrumentation and no cross-calibration is possible, as was the case for breaks between successive satellite ocean colour missions in the 1980s and 1990s. Any kind of discontinuity (from a statistical standpoint this is any event that results in a change in the mean and/or variance of a time series) dramatically increases the length of time series needed to detect a long-term trend [92].

Analysis of biological SO data for long-term trends has thus far been relatively rudimentary compared to the statistical techniques that have been applied to other geophysical time series. For example, change point analysis for detecting regime shifts have been only sparsely applied to biological SO data [95,96]. Another approach, optimal fingerprinting, is widely used in physical atmospheric and oceanographic applications [97,98,99], but putative long-term trends in biological SO data have not been tested using this methodology, or many of the others developed in climatology studies [100,101,102]. The slow uptake of statistical approaches is probably due in part to the aforementioned issues with the brevity of, and gaps and inconsistencies in, the time series, which seem to be more acute in biological than in physical SO datasets. Nevertheless, ideally a formal attribution study should be carried out before an observed trend is attributed to anthropogenic climate change, to exclude the possibility of mistaking a response to natural variability as a long-term trend.

The role for SOs here is clear: long-term, consistent, frequent observations of ocean biogeochemistry are required to make progress in understanding one of the primary challenges affecting the social and economic conditions of the human population—the impact of climate change on our oceans and its feedback into the whole Earth system.

### (b) New process understanding and new technology

Our ability to observe specific processes with SO platforms is developing hand-in-hand with new technology. Innovations in automated sampling technology are opening up new research avenues, thanks to the ever-increasing range of parameters that can be measured autonomously and improvements in the lifespan and reliability of the sensors.

Sustained monitoring of phytoplankton and zooplankton species information, including genetic information, is now possible [103,104,105]. A few examples illustrate the diversity of research already under way with this technology, such as: real-time monitoring of coastal waters for harmful algal blooms [106]; investigation of the seasonal dynamics of *Synechococcus* in relation to nitrogen supply [107]; examination of high spatial variability in zooplankton around upwelling fronts [108]; and genetic analysis of material from the CPR [109]. Future research topics exploiting this technology could potentially centre on the seasonal dynamics and species succession of the phytoplankton and zooplankton community at open-ocean time-series stations. At sites, such as the Porcupine Abyssal Plain, that include sediment traps and an upper-ocean mooring, community succession data could be explored in the context of variability in upper-ocean physics and links to deep carbon flux. In parallel, satellite-derived estimates of phytoplankton community structure have been developed that, although no consensus yet exists about the ‘best’ approach [110], may mature to the point that they could be used routinely (as is satellite chlorophyll or primary production data) to investigate large-scale variability. A recent modelling study suggests that climate change effects will be more pronounced at the phytoplankton population level (within functional groups) than in bulk primary production [111]. Therefore, monitoring species or ecotype composition may be an effective route to detecting long-term change in ocean biology.

Autonomous vehicles that sample with high vertical and/or temporal resolution, such as gliders and bio-Argo floats, have the potential to revolutionize our understanding of subsurface and (in the case of gliders) small-scale biophysical interactions. Gliders and bio-Argo floats may be equipped with temperature, salinity, chlorophyll fluorescence, oxygen, backscatter and light sensors and sample at vertical resolutions of up to approximately 0.5 m. In the case of gliders, approximately four dives (i.e. eight profiles) to 1000 m depth can be completed per day. While satellite ocean colour data have enhanced our understanding of daily variability in surface chlorophyll, gliders and floats have the potential to extend this knowledge by observing the subsurface phytoplankton populations and their evolution over sub-daily time scales. The lifespan of gliders and floats makes them suitable for studying seasonal variability in biophysical interactions in unprecedented detail, e.g. the processes surrounding the initiation of the phytoplankton spring bloom, a subject that is back in the spotlight and the subject of much debate (e.g. [112,113,114]). The high vertical resolution of sampling will also permit investigation into, for example, phytoplankton thin layers (e.g. [115]) and the evolution and erosion of the deep chlorophyll maximum. There is also great potential for continued improvements to optical nutrient sensors [116], in addition to future development of new microfluidic sensors, e.g. nutrients [117] or genetic analysis [118], suitable for long-term deployment on gliders, floats or fixed moorings.

New technology for SO platforms may also help us to address a currently large gap in our understanding of the ocean's carbon cycle: the remineralization of sinking organic carbon. Currently, we are unable to make direct autonomous measurements of most rate processes, including upper-ocean export and mesopelagic remineralization of organic carbon, leaving a large gap in our observing capabilities between primary production by phytoplankton at the surface and the carbon flux captured by deep sediment traps. However, this is a crucial issue for the global carbon cycle, as even small changes in the characteristics of mesopelagic remineralization can have a substantial influence on atmospheric CO_2_ concentrations [12]. Recently developed technology based on Argo floats that provides estimates of POC flux and information on the characteristics of sinking particles [62,90] has the potential to supply time series of data over many months on variability in carbon flux on sub-daily to seasonal time scales, frequent estimates of remineralization and high-resolution images of the sinking particles. Oxygen and nutrient sensors on floats and gliders can also be used to estimate net community production, which is equivalent to export under steady-state conditions [119,120]. Currently, we have very limited information on the temporal variability of upper-ocean carbon flux (except from the few ship-serviced time-series stations, such as BATS and HOT), which restricts our understanding of the coupling between surface productivity and export events. We also lack adequate data on the spatial and temporal variability in mesopelagic remineralization to the point that we have a relatively poor grasp of even the magnitude of variability [121], severely limiting our ability to define the important processes controlling remineralization rates. Knowledge of the type of sinking particles is also relevant to understanding controls on remineralization, as different kinds of aggregates, whole organisms and faecal pellets all have different carbon content, sinking speed and remineralization potential [122,123,124]. Thus, an improved ability to monitor carbon flux would lead to a step change in our understanding of a fundamental part of the global carbon cycle, which would also lead to more realistic parametrizations of remineralization in global biogeochemical models.

Improvements in the robustness of sensors and platforms will also allow us to expand SOs into harsh environments, such as polar regions. The Arctic is currently one of the most rapidly changing regions of the global ocean, with increasing temperatures driving rapid loss of sea ice. How this will affect the biology and biogeochemistry of the Arctic region is largely unknown. Sustained monitoring is restricted by the difficulties of operating in the Arctic, namely the harsh environment, remoteness and sea ice. However, developments that allow gliders and other autonomous vehicles to operate for sustained periods under ice or in ice melt conditions could overcome some of these issues. Despite the challenging conditions, intriguing biological phenomena have been observed in the Arctic, such as large phytoplankton blooms occurring along the seasonally retreating ice edge, beneath melt-ponded sea ice and in melt holes in perennial ice [125,126,127]. Future research priorities in the Arctic should centre on understanding whether these blooms are ubiquitous, their physical controls, e.g. the interaction between light and haline stratification, the marine ecosystem structure and how it changes seasonally as conditions progress from ice-covered to ice edge to open ocean. Importantly, we need to understand to what extent these blooms contribute to carbon export in the region and the potential of the Arctic to sequester carbon given its relatively shallow bathymetry. A particular issue with characterizing the Arctic's response to climate change is the lack of a baseline against which to compare observed variability. For example, we will be unable to determine whether a particular biological phenomenon has newly emerged, or how increased melting may alter an observed process. However, the combination of new technology that can reliably operate in difficult conditions and the pressing need to improve our understanding of even the basics of Arctic biology and biogeochemistry functioning, and therefore improve predictions of how it may continue to change in the future, should drive forward SOs of this vulnerable region.

### (c) Advances in analysis

The next breakthroughs in SOs will be enabled by advances in technology, but will also require advances in analysis techniques. SO programmes have, in the past, rarely been faced with dealing with large quantities of data, with the exception of satellite ocean colour data. However, with the advent of gliders and other autonomous systems that can sample quasi-continuously for weeks to months, the quantity of data will rapidly increase, bringing with it new challenges in analysing, visualizing and interpreting the data.

Some SO platforms, such as gliders or autonomous underwater vehicles, provide data that represent a mixture of spatial and temporal information. Disentangling the spatial from the temporal variability will require careful analysis and potentially input from other data sources, such as satellites. For example, a glider patrolling a fixed-point observatory may actually travel several kilometres in a day and therefore may unwittingly sample a mesoscale feature. If these data were to be interpreted as purely a time series, spurious conclusions could be drawn about the appearance and disappearance of intermittent features. A similar problem faces interpretation of data from any fixed-point observatory. Analyses such as the spatial decorrelation length scale, assessment of changing water mass characteristics and comparison with satellite data to provide spatial context will be necessary to distinguish spatial from temporal variability. Combining data streams, e.g. satellites, gliders and moorings, has the potential to be ‘greater than the sum of its parts’, providing a four-dimensional dataset; however, analysis and interpretation of the data will clearly be a challenge.

One area ripe for substantial advances is the combination of SO data with biogeochemical models. Physical–biological and trophic-level interactions deduced from an SO dataset can be investigated in a modelling framework, either by running a one-dimensional model in which forcing factors can be altered and the corresponding response assessed [128,129], or through exploration of a three-dimensional hindcast model run [87,88]. Provided a model reproduces the process or phenomenon of interest sufficiently well, its output could be used to plug gaps in the dataset, e.g. by supplying information on carbon export if that was not (or was not regularly) measured. Hindcast models can also be used as an extension of the SO dataset to investigate, for example, responses on decadal timescales. Finally, models can be used to make predictions of future climate change impacts. These can be used to separate the potential changes occurring due to climate change from those due to natural variability, and to provide a means of testing how different observational strategies may affect the detection of climate change effects.

Some SOs may soon enter the realm of ‘big data’, which will require advanced analytical techniques. The need for researchers skilled in programming and quantitative or statistical analysis is not unique to biological SOs, but is worth flagging here as a potential future obstacle to maximizing the novel results from SOs. Another route to maximizing exploitation of SO data is rapid open publication of datasets online, permitting meta-analyses of datasets from multiple SOs and greater uptake of the data by the wider community. Indeed, if an SO programme wishes to demonstrate the utility of its data, and so justify continued funding, it is in their best interests to ensure that data are readily available. This also requires specific support for data management, which is essential to ensuring long-term stewardship and easy access to quality-controlled datasets.

## 5. Limitations and challenges

SOs have made substantial contributions to our understanding of ocean biology and biogeochemistry and will continue to do so through maintenance of existing SOs and development of new platforms and sensors. There are, however, some challenges and limitations to SOs, many of which may be overcome with new technology, advances in analysis methods or, frankly, more investment.

There are many biological parameters and processes that are key to our knowledge of the marine ecosystem and its role in the Earth system that we cannot currently measure autonomously. Although ship-serviced time-series stations, such as BATS and HOT, can be a platform for these observations, the ability to make the measurements remotely from an autonomous platform would enable the coverage required to understand the large-scale distribution or variability. Undertaking SOs in remote or harsh environments also poses problems, including access to and servicing platforms, and poor operating conditions, such as rough seas, sea ice or heavy marine traffic, including fishing vessels. The development of new sensors with increased lifespan, reliability and accuracy, combined with the development of new SO platforms and integration with existing SOs, will, in time, address some of these limitations, although the future discovery of as yet unknown processes will undoubtedly continue to add to the list of parameters we would like to measure autonomously!

A persistent challenge for interpreting much SO data (with the exception of satellite ocean colour in some respects) is the convolution of spatial and temporal variability. As stated in §2, variability can be a blessing (in that it helps determine the controls on the process under investigation), but it can also be a curse. Prior to ocean colour satellites, the interpretation of time-series SO data in the light of a continually evolving mesoscale field proved difficult. Even with the benefit of satellite data to provide the spatial context, interpreting data from high-resolution samplers that move through both space and time (e.g. gliders) is extremely challenging. The temptation is to think of the SO data as a time series, but in many cases the data are actually a complex interplay of spatial and temporal variability [26,130,131]. Another concern, particularly with time-series stations, is evaluating to what extent the observations at that location are representative of a larger region. For example, are the observations made at BATS representative of the entire North Atlantic subtropical gyre, or only of the Sargasso Sea, or possibly even just a small corner of the Sargasso Sea? Repeat transects, such as the AMT programme, capture large-scale spatial patterns but can themselves suffer from a seasonal bias, e.g. cruises may only occur in spring. This brings with it two issues: first, our understanding of biological processes is limited to only one period, which may not be typical of the situation in the rest of the year; and second, multiple years of a repeat transect cannot necessarily be interpreted as a time series. Even though a series of cruises may occur at the same time each year, it is unlikely that the same point in the seasonal cycle is captured in each cruise, as temporal variability in ocean biogeochemistry is so large. In other words, a cruise on 1st April may encounter pre-bloom conditions in one year, the peak of the bloom in the following year, and post-bloom conditions in the year after that. Satellite ocean colour data can help to set the context in these situations by providing information on the broader spatial and temporal milieu.

An emerging issue with regard to detecting the influence of climate change on ocean biology is the longevity, consistency and completeness of SO data. Model analyses suggest that approximately 30–40 years of continuous data are needed to distinguish a climate change trend in primary production from the background natural variability [21,89]. Some SO programmes are now reaching (or have exceeded) this length; however, gaps or inconsistencies in the time series significantly increase the number of years of data needed to detect a climate change signal [92]. Gaps in a time series may arise due to technical failure of an instrument or platform, or due to the mothballing of an SO programme. Inconsistencies are caused by changes to sampling or analysis protocols, or a change in instrumentation without adequate cross-calibration. The advantages of maintaining a consistent time series are clear in, for example, the CPR survey, which has implemented the same protocols since 1948, providing a hugely valuable dataset. By contrast, the long gap and change in instrumentation in ocean colour satellites between the demise of the Coastal Zone Color Scanner (CZCS) in 1986 and the launch of SeaWiFS in 1997 makes direct comparison of the datasets fraught with difficulties [132,133]. The current threat of another gap in the ocean colour time series due to the demise of MERIS in 2012 and the now-aging MODIS instruments raises the possibility of a break in the dataset at a crucial juncture for the detection of climate change effects on ocean biology.

Finally, an ever-present challenge to SOs is securing long-term funding [134]. Sustained observing is slow science; it can take years or even decades before the full value and potential of the data is realized. There is also no doubt that SOs can be expensive, although when considering costs, the enormous quantity of data obtained by SOs should be borne in mind. For example, ocean colour satellites come with eye-wateringly large price tags; however, by the end of its mission, chlorophyll data from SeaWiFS had cost just 0.1 p per pixel. Long-term support of SOs should not just entail covering the basic maintenance and infrastructure costs. To fully exploit the rich datasets produced by SOs, considerable resources must also be put into the analysis, interpretation and dissemination of results, although this aspect of SOs can too easily fall by the wayside when resources get tight. There seems to be a perception among some funding agencies that we continue to maintain SOs out of inertia. This attitude must be guarded against by demonstrating the importance of the observations through rapid, community-wide exploitation and publication of the results.

## 6. Conclusion

Slow science, the kind that takes many, many years of consistent data collection, produces ground-breaking, deeply insightful and immensely valuable science. SO programmes have enabled some of the seminal hypotheses in biological oceanography to be first developed and then thoroughly tested. This illustrious history will undoubtedly lead in the future to more innovative results. The value of SOs to biological oceanography is hard to overstate: without them we would not understand even the basics of the spatial distribution of ocean productivity, the seasonal evolution of phytoplankton blooms, how the interactions from physics through to fish lead to variability in the ecosystem, or the magnitude of organic carbon reaching the deep ocean, to name just a few examples.

In the near future, SOs will be critical for our ability to detect climate change-driven trends in the marine ecosystem and as such their enormous value should be evident. However, with decreasing science budgets, we must remain vigilant to the perception that we maintain SOs out of inertia. We need to build the recognition that science originating from SOs is of high quality and increasingly relevant in a changing climate. Open access to rapidly calibrated and quality-controlled datasets will ensure exploitation by the broadest possible user community and rapid dissemination of results.

Development of new technologies is opening up new frontiers for biological SOs and with that comes the need to answer some potentially tough questions, such as: What are the scientific questions that we want our SOs to answer? Are our current SOs fit for that purpose? Are they measuring the right parameters in the right place at the right frequency to address the questions we want them to answer?

In a time of increasing pressures on the marine environment, SOs are central to understanding past, current and future changes in ocean biology and to monitoring future responses to climate change. With many SOs having now accumulated sufficiently long time series to quantify variability and trends, it would be frankly foolish to allow reductions in sampling or long gaps to occur. A primary concern for many (including the author) is the looming possibility of a gap in the satellite ocean colour record, which would introduce a break in a global, daily, spatially resolved, 16+ year time series. If the next global ocean colour sensor (OCLI on Sentinel-3) is not launched before MODIS-Aqua expires (currently 11 years old, 5 years older than its design lifetime), the gap could have serious consequences. The same is true of other SOs—if our dedication to maintaining them falters now, we could well be letting the opportunity to assess climate change effects on ocean biogeochemistry slip through our fingers.
